# Randomized controlled trial demonstrates novel tools to assess patient outcomes of Indigenous cultural safety training

**DOI:** 10.1186/s12916-023-03193-y

**Published:** 2024-01-09

**Authors:** Janet Smylie, Michael A. Rotondi, Sam Filipenko, William T. L. Cox, Diane Smylie, Cheryl Ward, Kristina Klopfer, Aisha K. Lofters, Braden O’Neill, Melissa Graham, Linda Weber, Ali N. Damji, Patricia G. Devine, Jane Collins, Billie-Jo Hardy

**Affiliations:** 1 Well Living House, Unity Health Toronto - St. Michael’s Hospital, 30 Bond Street, Toronto, ON M5B 1W8 Canada; 2 School of Kinesiology and Health Science, York University, 364 Bethune College, 4700 Keele Street, Toronto, ON M3J 1P3 Canada; 3 Inequity Agents of Change, Madison, WI 53714 USA; 4 Ontario Federation of Indigenous Friendship Centres, 219 Front Street East, Toronto, ON M5A 1E8 Canada; 5 Anti-Indigenous Racism Consultant, Nanaimo, BC Canada; 6 Womens College Hospital, Women’s College Hospital, 77 Grenville St, Toronto, TO M5B 2S1 Canada; 7 MAP Centre for Urban Health Solutions, St. Michael’s Hospital, Unity Health Toronto, 30 Bond Street, Toronto, ON M5B 1W8 Canada; 8 Department of Family and Community Medicine, University of Toronto, 500 University Ave, Toronto, ON M5G 1V7 Canada; 9 St. Josephs Heath Centre Family Medicine/Urban Family Health Team, 30 The Queensway, Toronto, ON M6R 1B5 Canada; 10 Credit Valley Family Health Team, 2300 Eglinton Avenue W Suite 105, Mississauga, ON L5M2V8 Canada; 11 Department of Psychology, University of Wisconsin - UW-Madison, 1202 W Johnson St, Madison, WI 53706 USA; 12 Vancouver, Canada; 13 Dalla Lana School of Public Health, University of Toronto, 155 College St Room 500, Toronto, ON M5T 3M7 Canada

**Keywords:** Indigenous cultural safety, Medical education, Racism in health care, Race bias, Health care disparities, Clinical trial, Health services research

## Abstract

**Background:**

Health care routinely fails Indigenous peoples and anti-Indigenous racism is common in clinical encounters. Clinical training programs aimed to enhance Indigenous cultural safety (ICS) rely on learner reported impact assessment even though clinician self-assessment is poorly correlated with observational or patient outcome reporting. We aimed to compare the clinical impacts of intensive and brief ICS training to control, and to assess the feasibility of ICS training evaluation tools, including unannounced Indigenous standardized patient (UISP) visits.

**Method:**

Using a prospective parallel group three-arm randomized controlled trial design and masked standardized patients, we compared the clinical impacts of the intensive interactive, professionally facilitated, 8- to10-h Sanyas ICS training; a brief 1-h anti-bias training adapted to address anti-Indigenous bias; and control continuing medical education time-attention matched to the intensive training. Participants included 58 non-Indigenous staff physicians, resident physicians and nurse practitioners from family practice clinics, and one emergency department across four teaching hospitals in Toronto, Canada. Main outcome measures were the quality of care provided during UISP visits including adjusted odds that clinician would be recommended by the UISP to a friend or family member; mean item scores on patient experience of care measure; and clinical practice guideline adherence for NSAID renewal and pain assessment.

**Results:**

Clinicians in the intensive or brief ICS groups had higher adjusted odds of being highly recommended to friends and family by standardized patients (OR 6.88, 95% CI 1.17 to 40.45 and OR 7.78, 95% CI 1.05 to 58.03, respectively). Adjusted mean item patient experience scores were 46% (95% CI 12% to 80%) and 40% (95% CI 2% to 78%) higher for clinicians enrolled in the intensive and brief training programs, respectively, compared to control. Small sample size precluded detection of training impacts on clinical practice guideline adherence; 100% of UISP visits were undetected by participating clinicians.

**Conclusions:**

Patient-oriented evaluation design and tools including UISPs were demonstrated as feasible and effective. Results show potential impact of cultural safety training on patient recommendation of clinician and improved patient experience. A larger trial to further ascertain impact on clinical practice is needed.

**Trial registration:**

Clinicaltrials.org NCT05890144. Retrospectively registered on June 5, 2023.

**Supplementary Information:**

The online version contains supplementary material available at 10.1186/s12916-023-03193-y.

## Background

Health care services routinely fail Indigenous populations, including in relatively affluent countries with public commitments to equitable service access [1–4]. The lack of due consideration of Indigenous social requirements in the design of domestic health care is an evident and persistent legacy of settler colonialism and the linked policies, systems, and structures that entrenched white supremacy, racial hierarchies, and the devaluing of Indigenous peoples, lives, and societies [5, 6]. Apologies by world leaders [6], governmental commissions and inquiries [7–10], and media documentation [11–13] have contributed to growing public recognition of anti-Indigenous racism in health care and anti-Indigenous colonial injustices, abuses, and harms more generally. Emerging recommendations for improving health care frequently include the implementation of training for clinicians [8] so they can better care for Indigenous patients, which in turn has contributed to rapid growth in the design and implementation of a variety of Indigenous cultural sensitivity, competency, and/or safety training programs and activities.

Evidence synthesis focused on understanding the impacts of Indigenous focused clinician training initiatives reveals varied approaches and evaluation measures that are almost exclusively learner focused as opposed to patient derived and oriented [14], even though clinician self-assessment is known to be poorly correlated with observational or patient outcome reporting [15, 16]. This gap in the assessment of patient care outcomes has been similarly noted across the broader domain of clinician anti-bias training [17].

Unannounced standardized patients (USPs) are a tool that can be used to assess patient facing communication, relationship skills, and clinical practice guideline adherence by health care professionals (HCPs) [18–21]. USPs are particularly relevant in clinical contexts that preclude the use of clinical record audits to assess equity in service provision due to gaps in reliable and consistent identification of equity deserving individuals in the medical record [19]. Moreover, USPs enable standardization of clinical and patient variables and mitigate challenges in observational study design linked to variability in the volume, predictability, and distribution of patients from equity deserving populations across provider practices [19]. With respect to performance assessment, USPs have been demonstrated to be better than HCP charting and comparable to researcher scoring of audio-recorded visits [22–24].

Gaps in evidence and health system staffing and funding constraints require decision-makers to make cost-effective decisions in their evaluation of the benefits of brief (and less costly) versus intensive Indigenous Cultural Safety (ICS) training for their physicians and staff. One approach that stands out among brief anti-bias training interventions is the brief (45 min) prejudice habit-breaking intervention [25–28]. While it has not yet been tested for patient-oriented outcomes, it has demonstrated sustained, socially relevant impacts [25–28]. The San’yas Indigenous Cultural Safety Training Program is a 10-h, eight-module professionally facilitated online program that has been delivered to > 170,000 health and social service professionals across Canada [29] and has been extensively evaluated [30]. In partnership with Drs. Devine, Cox, and the San’yas ICS program, our interdisciplinary team designed a randomized controlled trial (RCT) to evaluate the clinical impacts of an adapted version of the brief prejudice habit-breaking intervention and the intensive San’yas ICS training programs for academic physicians, nurse practitioners, and residents affiliated with several large teaching hospitals in or near the City of Toronto, Canada, using unannounced Indigenous standardized patients (UISPs) to assess clinical impact. The feasibility of using UISPs in this context was unknown. The primary objectives of this trial were therefore to compare the clinical impacts of intensive and brief ICS for health care providers (HCPs) and to assess the feasibility of new ICS evaluation tools, including UISPs.

## Methods

### Study design

We conducted a multi-site, three-arm, parallel group RCT. We randomized 58 health care providers to brief ICS, intensive ICS, or time-attention matched continuing medical education (CME) training (control regimen).

### Participants

We recruited academic physicians, resident physicians, and nurse practitioners (NPs) from 13 family medicine clinics and 1 emergency department across 4 large teaching hospitals in or near Toronto, Canada, between March 2018 and August 2021. Participants needed to identify as non-Indigenous and be committed to remaining in their current clinical setting for a minimum of 1 year post recruitment. We excluded anyone who had already completed a San’yas ICS training program. We also excluded physician leads at each site, who were unmasked to the study to facilitate its conduct.

Clinicians were recruited through presentations at staff meetings, followed by email invitations. Provided information included a description of the study as an RCT to evaluate the effectiveness of Indigenous cultural safety training and a description of the three study arms. An online survey platform, Qualtrics [31], facilitated study enrollment, including consent, baseline measures, and randomization.

### Randomization and masking

Participants were allocated equally (1:1:1) to one of the two interventions or the control arm independently of the research team using the randomization function of the Qualtrics platform [31]. Participants were not informed of the UISP visit and thus masked to this primary outcome measure. UISPs, research, and site team members were masked to participants’ study arm allocation except for the study coordinator, who facilitated enrollment in intervention and control training groups.

### Interventions

Both the brief and intensive interventions are designed to interrupt anti-Indigenous racism and draw on explicitly anti-racist and adult education pedagogies. The pedagogy of the intensive San’yas intervention is rooted in decolonizing critical race and transformative change theory. There are three purposefully sequenced content areas: history and current experiences of First Nations, Inuit, and Metis peoples; self-location; and cultural safety skills building. This content is delivered online with support from trained facilitators in a 10-h, eight-module format over 10 weeks. The version used in this trial was regionally adapted for Ontario (Additional File 1).

The brief anti-bias intervention is a 45-min interactive, computer-based education session focused on recognizing and addressing anti-Indigenous race bias followed up with two re-enforcement/reminder emails that ask questions about strategy usage at 6 and 8 weeks after the session. The version used in this trial (Additional File 2) was specifically adapted to focus on anti-Indigenous race bias from an existing training program that had been validated for other forms of bias [28]. This brief training was delivered in a computer lab with a researcher present. Adaptation included incorporation of Indigenous-specific scenarios and content and was supported by multiple study team members (WC, DS, BH, PD).

Participants randomized to the control arm were enrolled free of charge in their choice of the following accredited continuing medical education programs: the College of Family Physicians of Canada’s Self-Learning Program [32], Evidence-Based (EB) Medicine [33], or the Medical Knowledge Self-Assessment Program (MKSAP) [34]. These training programs were time-attention matched to the intensive intervention and did not include any content on anti-bias, anti-oppression, and/or Indigenous peoples.

We tracked participant completion of intervention and control trainings. Completion of trainings generated CME credits for participants that could be applied to meet requirements for maintenance of professional licensure.

Upon completion of the study and all associated measures, participants randomized to the brief intervention and control arms were provided free access to the San’yas Indigenous cultural safety education training program, since it was the ICS program with the strongest evidence support at the time of study design.

### Outcomes

Main outcome measures were focused on the assessment of the quality of care provided and clinical practice guideline adherence during UISP visits occurring 8–10 weeks post intervention. Quality of care was assessed using the Quality of Health Care Provider Relationship and Communication tool (Additional File 3). This 14-item scale assessed multiple domains of patient experience. The first ten questions assessed patient engagement and communication and were drawn from existing patient experience surveys developed and used to evaluate patient experiences in Canada and the UK [35–37]. In the absence of existing validated patient experience questions specifically assessing Indigenous cultural safety, we included a new question that drew from previous qualitative work [38] and a second question that was adapted from the Mothers on Respect Index [39]. Question 13 assessed respect [37] and Question 14, “Would you recommend this health care provider to family and friends?” has been used as an overall measure of patient satisfaction and was analyzed independently [40].

Two newly developed scales designed by clinicians on the research team assessed adherence to clinical practice guidelines during the UISP visits for NSAID renewal and pain assessment, respectively. The NSAID Renewal assessment scale and the Pain Management scale drew on existing clinical practice guidelines [41–44].

Secondary outcomes included pre- and 9–11 weeks post-intervention measures of anti-Indigenous race preference bias, using two scales assessing explicit bias (Modern Prejudiced Attitudes Towards Aboriginals, Motivation to Respond Without Prejudice) [45, 46] and The Indigenous Peoples in Canada—Implicit Association Test [47, 48].

### Sample size

We were not aware of any other HCP Indigenous race-bias reduction training intervention trials and recognized that this first study of its type would provide an opportunity to calibrate sample size requirements for future studies. We originally chose a sample size of 60 participants in each study arm or 180 total participants based on the study by Borkoff et al. [21], which used a comparable relational assessment scale to compare patient engagement and communication of physicians with male and female standardized patients presenting as possible candidates for knee arthroplasty. Drawing on the adjusted physician interpersonal scale scores in that study of 49% (95% CI: 45.0–53.0) and 63% (95% CI 59–67), we conservatively assumed a mean score for our 13-item relational score of 50% (since this is the prevalence that would require the largest sample) with a SD of 10%. Allowing for 20% participant attrition, we calculated that 180 participants would allow detection of a mean difference of 5.6% between study arms. Our target sample size was subsequently reduced to 60 participants total due to lower-than-expected enrollment rates from potential participant pools, which was exacerbated by the onset of the COVID-19 pandemic. This revised target sample size drew on Hochberg’s quasi-experimental evaluation of a six-session interactive communications training for surgical residents, that compared standardized patient assessment of pre- and post-training performance on five objective clinical structure case scenarios and demonstrated statistically significant changes for the question “Would you recommend this health care provider to family and friends?” with a sample size of 15 [40].

### Data collection

**Fig. 1:**
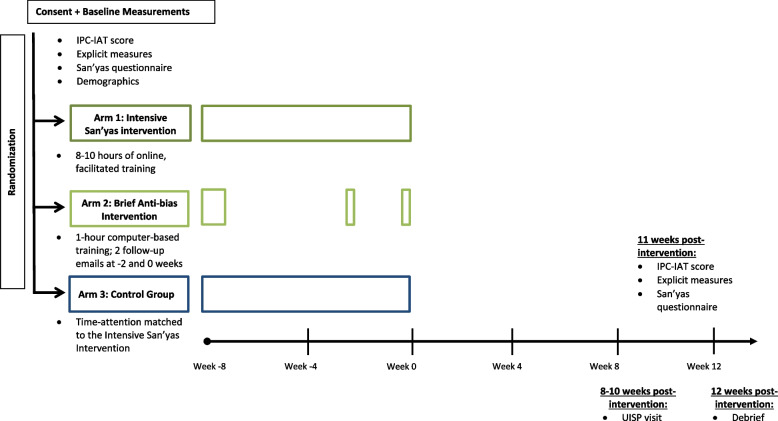
Study timelines

Primary outcome measures were scored by trained UISPs immediately following UISP visits, which occurred 8–10 weeks post completion of training and control interventions. Three male and six female professional middle-aged Indigenous actors without confounding health conditions were trained to perform and score one scenario, which involved an Indigenous patient presenting to study participants at family practice linked urgent care clinics or the emergency room with an acute flare of ankylosing spondylitis and requesting a renewal of their NSAID (Additional File 4). UISPs training included standardization of scoring using six mock video encounters applying this scenario that demonstrated varying levels of HCP competence. One week post UISP visit, participants received email reminders to complete secondary outcome measures using a provided link to the online Qualtrics platform. Two to 3 weeks post UISP visit, our research ethicist met with participants to disclose UISP visits, debrief, share a summary of their results, and provide an opportunity for study withdrawal or renewed consent for study participation (Fig. 1). Following initial training to standardize scenario presentation and assessment, UISPs attended regularly scheduled re-standardization sessions. When SARS-CoV-2 pandemic health service access protocols interfered with in-person UISP visits in March 2020, we pivoted to virtual visits, which required small modifications to the UISP scenario (Additional File 4).

### Statistical analyses

Overall patient experience compared the average item score for questions #1–13 across the three intervention groups using ANOVA. Given the potential for small cell sizes, Fisher’s exact test was used to examine potential differences in rates of the proportion of HCPs who were highly recommended (question #14). Similar ANOVA analysis strategies were used for the overall NSAID and Pain Management scales, which were scored out of 10 and 12, respectively. To preserve the benefits of randomization and to reduce bias, we applied an intention-to-treat approach to our analyses. To control for potential chance imbalances due to our relatively small study size, multiple regression analyses were used to control for the potential confounding effects of health care provider age, gender, and previous Indigenous experience. In the case of binary outcomes, multivariable logistic regression models were used. Secondary analyses of the Modern Prejudiced Attitudes Towards Aboriginals and Motivation to Respond Without Prejudice scales to assess anti-Indigenous race preference bias were analyzed using ANCOVA to control for baseline scores. All analyses were performed according to intention-to-treat principles at the two-sided, 5% significance level in the R environment (Version 4.2.1) [49].

### Ethics approval and Indigenous community advisory

The study was approved by Unity Health’s Research Ethics Boards (REB # 17–343). A temporary waiver of consent for UISP visits was obtained for study participants, as for design reasons they could not be informed of these visits during the initial informed consent. Disclosure and re-consent were obtained during the post UISP visit debrief by a research ethicist. This study was collecting data from non-Indigenous HCPs and therefore out of scope with respect to the formal application of Indigenous data sovereignty principles and protocols. We engaged the First Nations, Inuit, and Metis Community Advisory Panel (FNIM-CAP) of St. Michael’s Hospital-Unity Health Toronto (comprised of FNIM community members and FNIM and allied hospital staff) to act as study advisors, to inform study design and implementation, and help ensure there were no unintentional negative implications of the study for Indigenous people. No additional external data monitoring committee was deemed necessary given the minimal risk nature of the interventions.

We did not formally register the trial due to the exclusion of educational trials from clinical trial registration platforms at the time of design and the need to maintain participant masking of UISP visits.

## Results

**Fig. 2:**
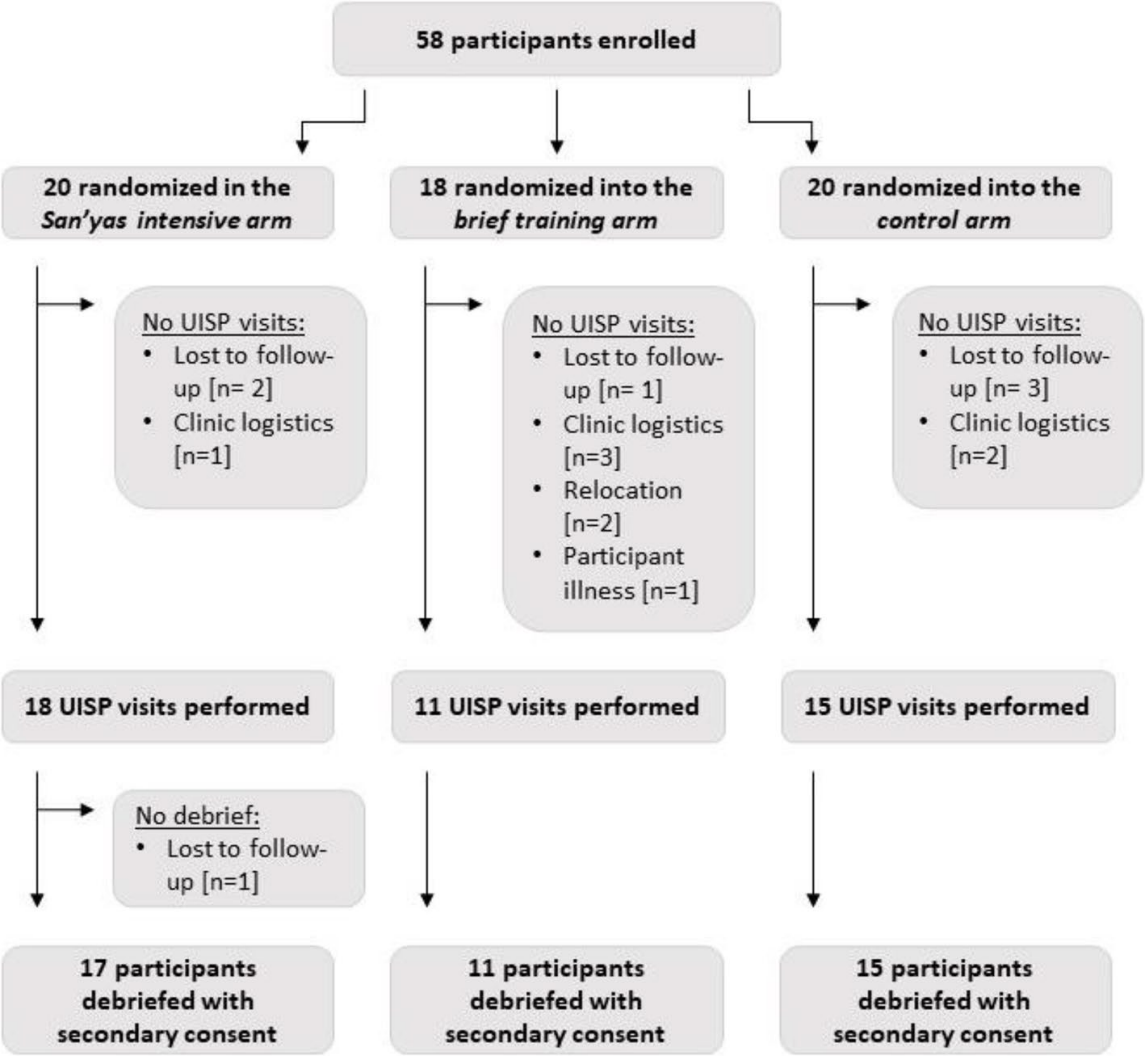
Participant enrolment and progression

A total of 58 subjects were enrolled and randomized. Due to the COVID-19 pandemic and other factors, including loss to follow-up and relocations, UISP visit data were not completed for 15 (26%) individuals, resulting in a final sample size of 43; 17 in the intensive intervention arm, 11 in the brief intervention arm, and 15 in the control (Fig. 2). The brief training regimen had the largest amount of subject attrition with 7 of 18 participants lost since randomization; 2 clinicians relocated, and 1 was ill and unable to be visited by an UISP. Figure 2 further details the trail flow for participants. The study ended when recruitment of clinicians was deemed saturated due to ongoing COVID-19 health system pressures and we had achieved 97% of targeted sample size. Rates of completion of the intervention and control trainings for the final sample were as follows: intensive Sanyas training (82%), brief anti-bias training (100%), and control training (100%). Three of the 17 participants in the intensive Sanyas training arm did not complete the full training. One of these participants completed 30% of the training and two participants completed 0% of the training. In keeping with our intention to treat approach, all 17 were included in the following analyses.

### Baseline characteristics

Table 1 includes baseline characteristics for the 58 participants who were randomized to the three intervention groups. We note that while most demographic variables were adequately balanced across groups, there was some potential evidence of chance imbalances in potential confounders, including age and previous participation in non-San’yas ICS training.

**Table 1:** Baseline characteristics of participants assigned to San’yas intensive training, brief anti-bias training, or control

	San’yas intensive training *N* = 20	Brief anti-bias training *N* = 18	Control *N* = 20
Age: Mean (SD)	44 (14)	38 (11)	42 (13)
Gender: (*N* (%))			
Male	6 (30%)	5 (27.8%)	5 (25%)
Female	14 (70%)	13 (72.2%)	15 (75%)
Racial/ethnic group			
Non-White	4 (20%)	7 (39%)	9 (45%)
White	16 (80%)	11 (61%)	11 (55%)
Professional designation:			
Staff	9 (45%)	9 (50%)	14 (70%)
Resident	10 (50%)	8 (44.4%)	6 (30%)
NP	1 (5%)	1 (5.6%)	0 (0%)
Professional practice			
Residents	10 (50%)	8 (44.4%)	6 (30%)
< 5 years	2 (10%)	1 (5.6%)	4 (20%)
5 to 10 years	1 (5%)	5 (27.8%)	1 (5%)
10 + years	7 (35%)	4 (22.2%)	9 (45%)
Department			
Emergency	3 (15%)	2 (11.1%)	2 (10%)
Academic FHT	15 (75%)	15 (83.3%)	17 (85%)
North York Community practice	2 (10%)	1 (5.6%)	1 (5%)
Previous Indigenous experience^a^	2.6 (1.0)	2.6 (1.3)	2.9 (1.4)
Participated in other cultural safety training^b^			
No	17 (85%)	15 (83.3%)	20 (100%)
Yes	3 (15%)	3 (16.7%)	0
Participated in other anti-oppression training			
No	17 (85%)	12 (66.7%)	18 (90%)
Yes	3 (15%)	6 (33.3%)	2 (10%)

*FHT* Family Health Team, *NP* nurse practitioner

^a^Likert scale out of 7

^b^Other training programs included non-evidence-based medical school content, seminars, and passive online training

### Effects of training

Unadjusted primary outcomes, including overall patient experience, proportion of highly recommended health-care provider, NSAID renewal, and pain management scores are presented in Table 2. Except for NSAID renewal, both the San’yas intensive and brief training regimens show potentially higher scores due to their respective interventions, though none of these improvements reach the traditional levels of statistical significance.

**Table 2:** Unadjusted analyses comparing the San’yas intensive training, brief anti-bias training, and control groups. Mean (SD), *N* (%)

	San’yas intensive training (*N* = 17)	Brief anti-bias training (*N* = 11)	Control (*N* = 15)	*P*-value^a^
Patient experience	4.52 (0.53)	4.57 (0.52)	4.22 (0.55)	0.1828
Highly recommended	10/17 (58.8%)	6/11 (54.6%)	4/15 (26.7%)	0.1979
NSAID renewal	6.31 (2.33)	8.27 (2.00)	7.33 (1.80)	0.0624
Pain management	8.94 (1.98; *N* = 16)	9.45 (1.92)	8.07 (2.34)	0.2421

^a^*P*-values are calculated using ANOVA or Fisher’s exact test, as appropriate

Multivariable adjusted analyses for the potential confounding effects of age, gender, and previous Indigenous experiences were used to further explore our results (Table 3). Clinicians in the intensive or brief ICS groups had higher adjusted odds of being “highly recommended” to friends and family by standardized patients compared to clinicians in the control group [OR 6.88 (1.17 to 40.45) and OR 7.78 (1.05 to 58.03), respectively]. Similarly, adjusted mean item patient experience scores (95% CI) were higher for clinicians enrolled in the intensive and brief training programs compared to control [0.46 (0.12 to 0.80) and 0.40 (0.02 to 0.78), respectively]. Both intervention groups show a clinically relevant improvement in this domain. There was no evidence of any statistically significant differences between groups on clinical practice guideline adherence for NSAID and pain management (Table 3). Full adjusted model results and results for secondary explicit and implicit anti-bias scale outcomes measures are included in Additional File 5. We note that there was no evidence of any statistically significant changes in these outcomes.

**Table 3:** Crude and adjusted analyses comparing the San’yas intensive training, brief training regimen, and control groups. Adjusted analyses control for health care provider age, gender, and previous Indigenous experience

	San’yas intensive training	Brief anti-bias training
**Overall patient experience**		
Crude	0.30 (-0.087, 0.68)	0.35 (-0.075, 0.78)
Adjusted	0.46 (0.12, 0.80)	0.40 (0.02, 0.78)
**Highly recommend**		
Crude OR	3.93 (0.88, 17.56)	3.30 (0.64, 17.16)
Adjusted OR	6.88 (1.17, 40.45)	7.78 (1.05, 58.03)
**NSAID renewal**		
Crude	-1.02 (-2.52, 0.48)	0.94 (-0.72, 2.60)
Adjusted	-0.82 (-2.36, 0.72)	0.61 (-1.13, 2.34)
**Pain management**		
Crude	0.87 (-0.66, 2.40)	1.39 (-0.30, 3.08)
Adjusted	1.26 (-0.24, 2.75)	1.54 (-0.15, 3.23)

All analyses are relative to the control group as the reference and 95% confidence limits are presented in parentheses

Logistic regression and odds ratios are used for analysis of highly recommended; all other models are regression-based

### Feasibility

With respect to the feasibility of UISPs as an ICS training evaluation tool in family practice and emergency care settings, we found that UISP methods were generally positively received by participants upon disclosure during post-visit debriefing, at which time, all participants with UISP visits (*n* = 43) re-consented to remain in the study. Additionally, none of the UISP visits (*n* = 43) were detected by participating clinicians prior to debriefing and UISP visit datasets were complete for main study outcomes, except for one missing pain management outcome. One unanticipated challenge with the UISP method, raised by a small number of participants and site leads, was the potential of the method to undermine work and trust relationships among participant clinical teams. This was linked to the need for on-site study collaborators, who were also part of participants’ clinical teams, to be unmasked to UISP visits so they could support their implementation. For this reason, two potential hospital sites declined to participate in study, and one participating clinic ended study recruitment of clinicians from their site early.

Scheduling of UISP visits was logistically complex, as it required the participant to be on shift in the emergency room or on rota to see urgent patients in primary care clinics. As a result, 37% (*N* = 16) of UISP visits occurred after the 8–10-week post-intervention time window. Additionally, we were unable to implement UISPs in smaller community clinics where there were only one or two clinicians (*N* = 2) or the reception staff were employed by the participant (*N* = 3), as these situations precluded masking participants to UISP visits.

## Discussion

In this trial, we found that brief anti-bias and intensive ICS training programs increase the likelihood that standardized patients would highly recommend trained clinicians to a friend or family member and improved UISP scoring of HCP relational quality. We also found that it is feasible to assess brief anti-bias and intensive ICS training impacts in clinical settings using UISPs.

While the use of standardized patients as a training and/or assessment tool is widespread in health care education [50], we could not find any studies that had previously applied UISPs to evaluate ICS training. By demonstrating the feasibility of UISPs as a tool for evaluation of ICS training, our study contributes to the advancement of outcome measures from clinician focused assessment of knowledge, attitudes, and self-reported behavioral changes towards the appraisal of observed patient care. This innovation is relevant not only to the rapidly growing field of ICS training for health and social service providers but also to the much larger domain of anti-bias and anti-oppression service provider training [17].

The successful retention of participants in our study after disclosure of and debriefing regarding UISP visits may have been linked to the skill of the individual conducting the debriefs, who was an experienced applied research ethicist. While delays in scheduling 37% of the UISP visits beyond the 10–12 week planned post-intervention window may have reduced the observed effectiveness of training interventions compared to control, this highlights the logistical complexity of scheduling UISP visits during the planned time-window and UISP implementation more generally. Voiced concerns regarding potential negative impacts on work and trust relationships among participant clinical teams due to the need for on-site staff support for UISP visits merit further validation. One potential remedy for this would be for UISP visits to be adapted as part of institution wide quality improvement initiatives which require or request HPCs permission to see UISPs as part of ongoing quality assurance.

While promising with respect to magnitude of impact, our primary outcome results regarding the clinical impacts of intensive and brief ICS training lacked statistical precision and were restricted to observed relational quality measures due to small sample size. While these findings represent one of the only examples of observed clinical impacts of ICS or anti-bias training versus control, a larger trial is required to verify these findings, further validate and refine UISP methods and linked assessment tools, and better understand the relational and clinical practice guideline adherence effects of the brief anti-bias and intensive San’yas ICS trainings. In addition to HCP age and gender, previous Indigenous experience as assessed by the “Amount of Previous Indigenous Experience” scale (Additional File 6) was an independent predictor of relational clinical outcomes and therefore controlled for in our adjusted models. Future trials could also contribute to a better understanding of how pre-existing participant knowledge, attitudes, and experiences contribute to training outcomes, which would in turn could support customized matching of ICS training programs to trainee educational needs.

Additional strengths of this study include the Indigenous leadership of the research team; the applied research partnership with the San’yas ICS program; the adaptation of the prejudice habit-breaking training to specifically address anti-Indigenous race preference bias; and the adaptation and testing of multiple Indigenous specific assessment tools including questionnaires and a novel Indigenous-White Implicit Association test. There was very good fidelity with respect to completion of the two intervention and control trainings. The 82% completion rate of the intensive Sanyas ICS training is consistent with actual completion rates in the field and our intention-to-treat analyses ensure that the attributable impacts of this intervention reflect this.

Further limitations included the challenges we encountered in recruiting already overloaded academic clinicians and residents, which were exacerbated by the SARS-CoV-2 pandemic and required a revision of our original sample size. SARS-CoV-2 additionally required a pivot to virtual UISP visits between March 2020 and September 2021. Only two NPs enrolled in the study limiting generalizability to this subgroup. We were inadequately powered for a clustered RCT design, which would have addressed site variability. Recruitment of participants through voluntary response likely contributed to a self-selection sampling bias towards participants who may have had less anti-Indigenous racism than uninterested clinicians. While this bias would have had a conservative impact on study findings if we assume that less racism would reduce the magnitude of the training effects, it is worth noting. Future interventions would be strengthened by the inclusion of those most in need of training. We were also unable to control for potential cross-contamination of control group participants by participant colleagues working at the same clinical site who were randomized to and had completed study interventions. While we are not aware of any literature quantifying impacts of this type of cross-contamination on the clinical behavior of HCPs, it is worth considering in future study designs. Finally, we were unable to prospectively register and publish the study protocol. We did reach out to clinicaltrials.gov in 2017 to prospectively register the trial, but were informed that since our primary outcomes were measures of clinician performance and not patient outcomes, we did not qualify for trial registration. We were additionally unable to publish the protocol as to do so would have potentially unmasked participants to UISP visits and educational studies appeared to be excluded from the clinical RCT registration sites with which we were familiar. We agree with Masters that the creation of a registration site for medical education trials is an ethical necessity and are pleased that clincialtrials.gov accepted our protocol retrospectively in 2023 for registration and publication [51].

Despite growing acknowledgement, calls for action, and new training initiatives, there is very little evidence demonstrating tangible reductions in Indigenous health inequities. In fact, the SARS-CoV-2 pandemic contributed to a global widening of multiple Indigenous/non-Indigenous health disparities, including increased health service access barriers [52, 53]. In this context, there is a pressing need to better equip health care decision makers with clinical evidence as they weigh investments in Indigenous cultural safety training and linked recommendations regarding health systems transformations such as adequate resourcing and the advancement of Indigenous governance and Indigenous models of health and healing [7–10]. It is critical that public commitments to address Indigenous health inequities are translated into actions informed by evidence and recommendations from Indigenous leadership. “Cherry-picking” clinically undemonstrated ICS or anti-bias clinician training and implementing such trainings without accompanying robust organizational and system level transformation activities is common, but not evidence-based or aligned with existing policy and legal commitments [7–10].

We can and must do better. Evaluation standards for ICS trainings that include objective assessment of patient-oriented clinician training outcomes are essential to ensure trainings are improving Indigenous patient care. Moreover, ICS training needs to be linked to cross-institutional and system level initiatives that actively disrupt the differential provision of social opportunities and resources to settler populations over First Peoples and advance Indigenous governance and management of Indigenous affairs [6]. Isolated HCP ICS training initiatives represent a grossly insufficient health system response to domestic and international evidence and recommendations regarding the advancement of Indigenous health equity and social justice [7–10, 54].

## Conclusion

This RCT has demonstrated that UISPs are a feasible and effective tool to measure and advance the patient focused clinical impacts of ICS training and that brief anti-bias and San’yas intensive ICS trainings have positive potential impacts on simulated patient recommendation of clinician and patient experience compared to control. There is still much work to be done. A larger trial is needed to further ascertain impact of these trainings on clinical practice. We envision a robust offering of ICS trainings for HCPs that can be matched to learner needs and combined to form individualized life-long learning curricula, all of which have been demonstrated to positively impact clinical care for Indigenous patients and their families. ICS training must also be embedded in larger institutional and system change strategies that are addressing the full spectrum of recommendations for advancing the social value of health services for Indigenous populations. Addressing anti-Indigenous racism and inequities in health systems is a complex and multi-faceted challenge that requires individual level behavior change, organizational remodeling, and substantive system level social change. It is our hope that clinical, research, and health care decision-making colleagues will join us in advancing the global shift towards comprehensive, cross-cutting, and linked investments at the HCP, organizational and systems levels to tangibly enhance the social value of health services for First Peoples.

### Supplementary Information

**Additional file 1.** Intensive San’yas Intervention Summary.

**Additional file 2.** Brief Anti-Bias Intervention Summary.

**Additional file 3.** Unannounced Indigenous Standardized Patient (UISP) Data Collection Tools.

**Additional file 4.** UISP Scenario.

**Additional file 5.** Supplemental Adjusted Model Results for Primary Outcomes and Supplemental Tables and Results for Secondary Explicit and Implicit Anti-Bias Outcomes.

**Additional file 6.** Consort Checklist.

**Additional file 7.** Amount of Previous Indigenous Experience scale.

## Data Availability

The datasets generated and/or analyzed during the current study are not publicly available due to small numbers and the need to protect participant confidentiality. Questions regarding the datasets and/or requests for additional analyses can be addressed by contacting the corresponding author.

## Abbreviations

ANCOVA: Analysis of covariance
ANOVA: Analysis of variance
CI: Confidence interval
CME: Continuing medical education
COVID-19: Coronavirus disease of 2019
EB: Evidence-based
FHT: Family Health Team
FNIM: First Nations, Inuit, and Métis
FNIM-CAP: First Nations, Inuit, and Métis Community Advisory Panel
HCP: Health care professional
ICS: Indigenous Cultural Safety
MKSAP: Medical Knowledge Self-Assessment Program
NP: Nurse practitioner
NSAID: Non-steroidal anti-inflammatory drug
OR: Odds ratio
RCT: Randomized control trial
REB: Research Ethics Board
SARS-CoV-2: Severe acute respiratory syndrome coronavirus 2
SD: Standard deviation
UISP: Unannounced Indigenous standardized patient
USP: Unannounced standardized patient
