# Supplementary material for: Randomized controlled trial demonstrates novel tools to assess patient outcomes of Indigenous cultural safety training
Source: BMC Med. 2024 Jan 9;22:3. doi: 10.1186/s12916-023-03193-y (PMC10775432; doi:10.1186/s12916-023-03193-y)

**Additional File 1 –** ***Intensive San’yas Intervention* Summary**

The *Intensive San’yas Intervention* is a self-paced, online, and facilitated educational intervention that was co-founded and created Dr. Cheryl Ward while working at the Provincial Health Services Authority in British Columbia, Canada. To develop the training that was adapted for the purpose of this study, San’yas partnered with regional Indigenous health leaders and educators in Ontario. (29) The intervention’s key learning goals are to improve participant knowledge, self-awareness, and skills to work more safely and effectively with Indigenous people. The core health training also supports participants to practice self-reflection about the role they can play in support of organizational and systemic change to uproot anti-Indigenous racism. (29) The intervention’s pedagogy is rooted in critical race theory, postcolonial theoretical perspectives, and transformative learning theories, which are embedded within Indigenous epistemologies. (30) The Ontario training for core health competencies builds on foundational aspects of cultural safety and covers topics of: colonization in Canada; the impacts of racism, discrimination, and stereotyping on Indigenous patients in healthcare; the social and structural determinants of Indigenous health; Indigenous health inequities; and taking personal and organizational action against anti-Indigenous racism. The San’yas intervention is explicitly designed as a systems-level response to address racism and discrimination and as a pathway to health equity for Indigenous people. The course meets the accreditation criteria of the College of Family Physicians of Canada, the Royal College of Physicians and Surgeons of Canada and the Canadian College of Health Leaders.

The San’yas facilitation team of Indigenous and non-Indigenous educators guide cohorts of learners through facilitated, interactive, and self-paced modules. (30) The online classroom environment makes use of discussion board activities, reflective journaling, and other activities to encourage the active engagement of learners. (30) Facilitators provide learners with both standardized activities and personalized, one-on-one engagement to focus learners on critical self-reflection, power dynamics, and inequities embedded within the policies and practices of health services. (30) The facilitators’ skills and strategies to support learners’ as they experience a range of emotions and to shift their focus on racism at the individual level to the systemic level are paramount to the success of the intervention. These facilitation strategies guide learners to being identifying key areas for personal and organizational transformation. (30) San’yas seeks to create a safe learning environment for diverse learners, and utilizes strengths-based, trauma- and violence-informed, and culturally respectful approaches to education. (30)

*Training Screenshots*


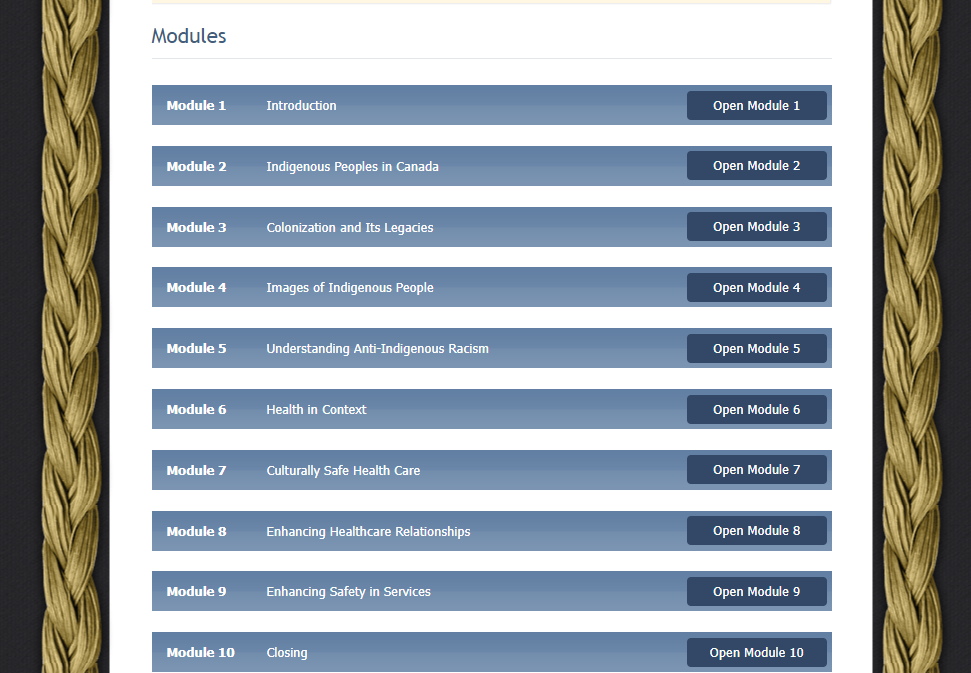

Supplement: Supplementary file 1 — Additional file 1. Intensive San’yas Intervention Summary. [file 12916_2023_3193_MOESM1_ESM.docx]
