# Supplementary material for: Randomized controlled trial demonstrates novel tools to assess patient outcomes of Indigenous cultural safety training
Source: BMC Med. 2024 Jan 9;22:3. doi: 10.1186/s12916-023-03193-y (PMC10775432; doi:10.1186/s12916-023-03193-y)
Supplement: Supplementary file 2 — Additional file 2. Brief Anti-Bias Intervention Summary. [file 12916_2023_3193_MOESM2_ESM.docx]

**Additional File 2 – *Brief Anti-Bias Intervention* Summary**

The *Brief Anti-Bias* *Intervention* is an Indigenous adaptation of the “Prejudice Habit-Breaking” intervention that has been extensively evaluated and shows long-term reduction in participant bias. (25-28) As by Cox et al. (28) the core principles of the computerized intervention, originally designed to address anti-Black bias, can applied to any stereotyped group. The “Generation 2” habit-breaking intervention (28) maintains the same fundamental design as the previous generation (25-27), and specifically trains participants to advance the following learning objectives:

1. Understanding the origins of stereotypes and biases and framing them as “habits”
2. Learning that stereotype and bias habits can be changed over time through individual Motivation, Awareness, Strategies, and Effort
3. Understanding the harms of unintentional bias
4. Using evidence-based strategies to reduce bias

Study team members, including experts from ICS Ontario and San’yas, PHSA (DS, JC) and one of the developers of the brief Anti-Bias Intervention (WC) adapted the intervention to reflect the Indigenous context in Canada, while maintaining its overall framework and anti-bias strategies. The adaptations made by the study team included: using new training scenarios that matched the experiences of Indigenous people in Canada; referencing relevant evidence from anti-Indigenous racism research; and utilizing a new Implicit Association Test for Indigenous Peoples in Canada (47,48).

The overall implementation of the *Brief Anti-Bias Intervention* remained the same as previous iterations of the “Prejudice Habit-Breaking” intervention. Participants were required to attend a supervised training session, where the computerized intervention was delivered under the supervision of a research staff member. Each training session lasted approximately 45 minutes and could not be interrupted. The intervention applied relevant Indigenous examples and Canadian context to explore the learning objectives listed above, and finished by exposing participants to five evidence-based anti-bias strategies (25-28):

1. Recognizing stereotypic responses (internal and societal), labeling them as stereotypes, and replacing them with non-stereotypic responses
2. Imagining examples of people who counter commonly held stereotypes
3. Individualizing others according to their personal, rather than stereotypic, characteristics
4. Taking a first-person perspective of a member of a stereotyped group
5. Increasing contact with members of the stereotyped group

Consistent with “Generation 2,” (28) participants were emailed a follow-up survey on their anti-bias strategy usage following completion of the training session. These surveys were delivered 6- and 8- weeks following the training session and reminded participants about the five anti-bias strategies, and then asked them to count the number of times they had used each of strategies over the previous weeks. Each of these surveys took approximately 5-10 minutes to complete, which brought the total length of intervention activities to nearly 1-hour.

**Brief Anti-Bias Training 6- and 8- week strategy usage follow-up survey**

We would like to understand more about people's use of the techniques that we showed you during the lab session you completed. In the following questionnaire, you will be presented with a brief description of each strategy that you learned.

Q 1) Stereotype replacement

When a stereotypic response has been detected, this technique involves labeling that response as stereotypical, evaluating the situation that generated the response, and replacing the response with a non-stereotypic response. This technique can be applied either to stereotypes that one detects in oneself or in society.

Over the past few weeks, how many times have you used this technique?

o 0 times

o 1

o 2

o 3

o 4

o 5

o 6

o 7 times or more

Q 2) Thinking of counter-stereotypic examples

This technique involves thinking of an example of someone, either a famous person or personally known, that counters a stereotype that one has detected in oneself or in society.

Over the past few weeks, how many times have you used this technique?

o 0 times

o 1

o 2

o 3

o 4

o 5

o 6

o 7 times or more

Q 3) Individuating (instead of generalizing)

Stereotyping involves applying the same set of characteristics to all members of a group on the basis of their group membership. Rather than generalizing across group members, people can individuate them, going beyond race and attending to personal characteristics.

Over the past few weeks, how many times have you used this technique?

o 0 times

o 1

o 2

o 3

o 4

o 5

o 6

o 7 times or more

Q 4) Perspective taking

This technique involves taking the perspective of an Indigenous person in the first person to see how it would feel to be judged based on stereotypes.

Over the past few weeks, how many times have you used this technique?

o 0 times

o 1

o 2

o 3

o 4

o 5

o 6

o 7 times or more

Q 5) Seeking opportunities for contact

This technique involves modifying one's environment by seeking interactions with Indigenous people or one's visual environment by watching movies or TV shows that portray Indigenous people in non-stereotypic ways.

Over the past few weeks, how many times have you used this technique?

o 0 times

o 1

o 2

o 3

o 4

o 5

o 6

o 7 times or more
