# Supplementary material for: Randomized controlled trial demonstrates novel tools to assess patient outcomes of Indigenous cultural safety training
Source: BMC Med. 2024 Jan 9;22:3. doi: 10.1186/s12916-023-03193-y (PMC10775432; doi:10.1186/s12916-023-03193-y)
Supplement: Supplementary file 3 — Additional file 3. Unannounced Indigenous Standardized Patient (UISP) Data Collection Tools. [file 12916_2023_3193_MOESM3_ESM.docx]

**Additional File 3 - Unannounced Indigenous Standardized Patient (UISP) Data Collection Tools**

**Unannounced Indigenous Standardized Patient Scoring Tool:**

**Quality of Health Care Provider Relationship and Communication**

**Thinking about the healthcare provider you saw during your visit today . . .**

**1. How would you evaluate the amount of time that the person gave you?**

- **Very good** (more than 15 minutes)
- **Good** (10-15 minutes)
- **Fair** (8-9 minutes)
- **Poor** (5-7 minutes)
- **Very poor** (less than 5 minutes)

**2. How would you evaluate the way the person listened to you during the visit? Markers of attentive listening include**: maintained eye contact, posture was leaning towards you, was not distracted, positive prompting and reinforcement (example: go on, yes I understand, tell me more), clarifying, summarizing what you are saying, probing questions.

- **Very good** (all of the markers)
- **Good** (eye contact, not distracted, positive prompting, summarizing, probing questions)
- **Fair** (eye contact, not distracted, positive prompting)
- **Poor** (no eye contact, some prompting)
- **Very poor** (no eye contact, distracted, doesn’t remember what you are saying)

**3. How would you evaluate the way the person involved you in decisions about
your care? Markers of involved decision making:** *differing to your lived experience* (what works best for you, what have you done before that you would like to see happen again); *checking to see if treatment options are affordable* (can you take time off of work, do you have coverage, would you prefer a generic drug instead of the name brand); *shared decision making* (this is the plan I propose does this work for you, here are some of the options we have moving forward what would you prefer); *consideration of your personal goals* (what would you like to see happen moving forward, how can I help you cope with this moving forward)

- **Very good** (all of the markers)
- **Good** (differing to lived experience, checking affordability, shared decision making)
- **Fair** (differing to lived experience, shared decision making)
- **Poor** (no consideration of lived experience, given some options)
- **Very poor** (no consideration of lived experience, no options provided

**4. Did he or she really find out what your concerns were? Main concerns: pain, heartburn, traditional medicine**

- **Yes, completely** (pain, heartburn, traditional medicine)
- **Yes, mostly** (pain, heartburn)
- **Yes, a little** (pain)
- **No, not really** (did not understand the intensity of pain)
- **No, not at all** (did not understand I was in pain)

**5. Did he or she let you say what you thought was important? Important: including traditional medicine in your care moving forward**

- **Yes, completely** (able to talk about traditional medicine and given support to include it)
- **Yes, mostly** (able to talk about traditional medicine, but not probed about how to include it)
- **Yes, a little** (mentioned traditional medicine)
- **No, not really** (mentioned traditional medicine and not met with receptive feedback)
- **No, not at all** (mentioned traditional medicine and met with dismissal)

**6. Did he or she take your health concerns very seriously?**

- **Yes, completely** (acknowledged pain, was empathetic, provided solutions to address them)
- **Yes, mostly** (acknowledged pain, was empathetic)
- **Yes, a little** (acknowledged pain)
- **No, not really** (acknowledged pain but didn’t believe it was as bad as you thought)
- **No, not at all** (completely dismissive of pain)

**7. Was he or she concerned about your feelings? Markers of empathy:** *Inviting exploration of unexpressed feelings* (you look like you would like to say, what’s on your mind, I see that you are having a thought), *effectively acknowledging these feelings* (I hear you, that is completely understandable, I can only imagine how difficult it must be)

- **Yes, completely** (expressed explicit acknowledgement of feelings and allowed you to elaborate)
- **Yes, mostly** (acknowledged feelings, some elaboration)
- **Yes, a little** (acknowledged feelings but did not probe)
- **No, not really** (acknowledge some feelings very briefly)
- **No, not at all** (didn’t acknowledge feelings, focused on symptoms)

**8. Did he or she explain your condition and treatment in a way you could understand?** Clear and simple language, spoke at a normal pace (wasn’t rushing through), asked if you needed clarification, no medical jargon - overall you are able to repeat the prescription you got, the symptoms to look out for and your overall diagnosis

- **Yes, completely** (normal pace, clear and simple language, able to repeat 3 items)
- **Yes, mostly** (rushed but simple language, able to repeat 3 times)
- **Yes, a little** (rushed, with some medical jargon, but able to repeat 3 items)
- **No, not really** (rushed, medical jargon, can repeat 2 out of the three items)
- **No, not at all** (rushed, medical jargon, unable to ask any questions, can’t repeat the 3 items)

**9. Did he or she give you clear instructions about symptoms to watch for and when to seek further care or treatment? Articulation of all the GI symptoms: blood in stool, black or tarry stool, vomiting blood, abdominal pain**

- **Yes, completely** (all symptoms are clear and a plan is in place to come back)
- **Yes, mostly** (some symptoms are clear and a plan is in place to come back)
- **Yes, a little** (you know that you have to look out for GI symptoms, but unclear on which ones and you know where to go in case of an emergency)
- **No, not really** (you don’t know what symptoms to look out for, but you know if you are not feeling better to come back or go to the ER)
- **No, not at all** (you don’t know what symptoms to look out for or where to go in case of an emergency)

**10. Did he or she discuss with you your main goals or priorities in caring for
your condition? Main goals and priorities include:** integrating traditional medicine into your care moving forward, being able to get back to your daily activities and manageable pain levels.

- **Yes, completely** (you were able to have a conversation about how to go about getting traditional medicine – even if the doctor didn’t know they made an effort to support you moving forward.)
- **Yes, mostly** (able to discuss managing pain and returning to daily activities, a little bit about the traditional medicine)
- **Yes, a little** (discussed pain management, nothing else)
- **No, not really** (did not speak to goals or priorities but prescribed solutions for pain management)
- **No, not at all** (no conversation about any of the three things)

**Overall, during the encounter with the healthcare provider:**

**11. …I felt comfortable and able to be myself as an Indigenous person.**

- 1. **Strongly disagree** (immediately stereotyped, did not feel comfortable bringing up the use of traditional medicine, or asking to include it in my care)
  2. **Disagree**
  3. **Neither**
  4. **Agree**
  5. **Strongly agree** (absence of stereotyping, felt comfortable bringing up )

**12. …I felt that I was treated poorly by the healthcare provider because I am Indigenous. Overall, the degree to which you felt you were stereotyped.**

1. **Strongly disagree** (was not stereotyped and was treated very well)
2. **Disagree**
3. **Neither**
4. **Agree**
5. **Strongly agree** (was immediately stereotyped based on Indigenous specific stereotype around pain medication, narcotic seeking behaviors, alcohol use and smoking)

**13. …I felt that I was treated with respect and dignity. Markers of respect and dignity:** privacy (if you were asked to change the room was vacated, you were able to keep your undergarments on), consent (can I touch your abdomen, can I your back, this is what I will do next, you will feel my hands here), greetings (addressed by your first and last name, asked for pronunciation), absence of patronizing language, your Indigenous heritage was respected (asking where you’re from, if you have managed to find supports in Toronto)

1. **Strongly disagree** (none of the criteria were met)
2. **Disagree**
3. **Neither**
4. **Agree**
5. **Strongly agree** (all the criteria were met)
6. **Would you recommend this health care provider to family or friends?:**

1. not recommend;

2. recommend with reservations;

3. recommend;

4. highly recommend.

1. **Additional process notes about the encounter**

---------------------------------------------------------------------------------------------------------------------------------------------------------------------------------------------------------------------------------------------------------------------------------------------------------------------------------------------------------------------------------------------

**Unannounced Indigenous Standardized Patient Scoring Tool:**

**Adherence to Clinical Standards of Care**

Standardized Patient ID #: ___________________

HCP Participant ID #: _____________________

Date of Encounter: _________________________

1. **NSAID prescription renewal request – total score up to 10 points**

- Needs improvement**: 5 or less**
- Acceptable**: 6-7**
- Meets standard: **8 or more**

1. Prescription of non-selective NSAID that is not diclofenac and Cox 2 inhibitors (due to CVD risk – borderline HTN/smoker) – up to 2 points

- 0 points: no prescription
- 1 point: prescription renewal for Diclofenac
- 2 points: prescription for non-selective NSAID other than Diclofenac and Cox 2 inhibitors

1. Prescription for less than 30 days with no repeats – up to 2 points

- 0 points: no prescription or prescription for greater than 30 days with repeats
- 1 point: prescription for 30 days with repeats
- 2 points: prescription for less than 30 days with no repeats

1. Adequate gastroprotection – 2 points

- 0 points: no conversation about gastroprotection
- 1 point: acknowledgement of need for gastroprotection but no clear options provided
- 2 points: prescription for gastroprotective agent and/or conversation about protective factors

1. Discontinue aspirin – 2 points

- 0 points: no mention of aspirin discontinuation
- 1 point: discontinue aspirin
- 2 points: discontinue aspirin with explanation about why/how to proceed in the future

1. Warn patient to discontinue NSAID and seek immediate f/u if any GI symptoms – 2 points

- 0 points: no follow up or discharge instructions specific to GI symptoms provided
- 1 point: discontinuation of NSAID but GI symptoms not properly articulated
- 2 points: clearly articulated discontinuation of NSAIDs and follow up if GI symptoms occur

1. **Assessment and management of acute pain – total score up to 12 points**
   - Needs improvement: **7 or less**
   - Acceptable: **8-9**
   - Meets standard: **10,11,12,**
2. Intensity of pain is documented using a numeric (0–10) or descriptive (mild, moderate, severe) rating scale. Up to 2 points

- 0 points: no scale is provided
- 1 point: scale is provided but not explained
- 2 points: scale is provided and explained
- ****For actors: MD may have different ways of asking about pain, ultimately, the purpose is ensuring that the MD appropriately captured the intensity of the pain.*****

1. Impact of the pain on daily activities is acknowledged and addressed (unable to work, disrupted sleep – examples: Clinicians acknowledges pain – ie. coping with that that must be difficult; that sounds terrible etc Addressing pain – add – writes a note for being off work or modified work duties) . Up to 2 points

- 0 points: pain on daily activities is not acknowledged
- 1 point: pain is acknowledged but is not addressed
- 2 points: pain is acknowledged and addressed (examples: coping strategies, referral to physiotherapy, discussion of what has helped in the past)

1. No opioids are prescribed – up to 2 points

- 0 points: prescription of opioids

- 2 points: no prescription of opioids

1. Patient feels adequately informed and knowledgeable about pain management – up to 2 points

- 0 points: patient does not feel knowledgeable about pain management (side effects, frequency of usage, dosage, red flags and when to return to clinic or ED)
- 1 point: patient understands some of the criteria but not all
- 2 points: patient was provided with all the above information in a clear manner

1. A follow-up plan is in place (ie f/u with family doctor within 1 month – sooner if pain not better) – up to 2 points

- 0 points: no follow up plan in place
- 1 point: follow up plan addressed but vague (“follow up with your GP”)
- 2 points: follow up plan in place and clear (“see GP in 3-4 weeks, sooner if pain is not improving”)

1. Physical examination performed – up to two points

- 0 point: no exam performed
- 1 point: musculoskeletal or abdominal exam only
- 2 points: musculoskeletal and abdominal exam performed.
