# Supplementary material for: Randomized controlled trial demonstrates novel tools to assess patient outcomes of Indigenous cultural safety training
Source: BMC Med. 2024 Jan 9;22:3. doi: 10.1186/s12916-023-03193-y (PMC10775432; doi:10.1186/s12916-023-03193-y)
Supplement: Supplementary file 4 — Additional file 4. UISP Scenario. [file 12916_2023_3193_MOESM4_ESM.docx]

**Additional File 4 – UISP Scenario**

The goal of this simulated clinical encounter is to assess the health care provider’s relational and clinical decision-making performance.

Relational performance will be assessed using a 14 point scale derived existing from patient experiences tools.

Clinical decision-making performance will focus on clinical practice guidelines adherence with respect to:

- Management of prescription request
- Assessment and management of acute pain

**Scenario Summary**

The patient’s feelings, ideas, and expectations of the visit, as well as an acceptable approach to management, are detailed in the case description, assessment tools and linked marking schemes.

**Background**

You suffer from ankylosing spondylitis, a form of arthritis that causes your lower back to flare up in pain. You have been dealing with it for 10 years. You have been experiencing increased stiffness and pain in your lower back for the last 2 months. It has been getting worse and is particularly bad in the morning. You went to a walk-in clinic close to work 3 days ago because the pain was so bad, you couldn’t wait to get an appointment with your family doctors. The walk-in doctor told you to take Extra Strength Advil three times a day for the next week and follow up with your family doctor if the pain persists. He also gave you a requisition for bloodwork and x-rays which you got done right away. The pain in your lower back has been persistent, but the Advil doesn’t seem to be helping and now you are experiencing heartburn.

**Affect**

You have pain in your lower back and you can’t sit. If asked, the pain is 7 out of 10 (attending physician/NP must explain scale before you assign a number) on the pain scale. You show discomfort in your face and body movements, so bad that you cannot sit. You are very stiff.

**Attire**

Casually dressed as you did not go into work today. May wear track pants and sweat shirt as dressing is painful. Also some type of tote to keep the empty Diclofenac bottle and any other props in.

**Opening line:** “I’m having a lot of pain in my lower back. It started 2 months ago, but has gotten really bad over the last two days. It feels like my ankylosing spondylitis is flaring up.”

**Prompt:** (bring up sometime later in the encounter, but only with attending physician/NP and only if you feel safe and not judged because of your Indigeneity) would you happen to know of an Aboriginal health centre where I could access a traditional healer to get some traditional medicine that helped my symptoms in the past.

**Case Description**

**WHO ARE YOU**

**Name: Tom or Theresa Whitford**

**DOB: December 2^nd^, 1969 (current age 48)**

**Born in: Calgary** You are of First Nations descent (Cree, Saddle Lake FN – grew up in Calgary).

**Moved to Toronto:** mother moved to Toronto to be closer to the family of her second husband – your step-dad

**Address: catchment area needs to match**

**Job:** Bank Manager

**HISTORY OF THE PROBLEM**

**Ankylosing Spondylitis**

As described above, you were diagnosed with Ankylosing Spondylitis 10 years ago in Calgary by Dr. Barnabe, a rheumatologist. You had been referred to her by Dr. Lindsay Crowshoe, a family physician at the Aboriginal health clinic in Calgary who you went to see after experiencing progressive lower back stiffness and pain for several months. Dr. Barnabe prescribed a course of Diclofenac and your symptoms resolved quickly. You had 2 or 3 flares over the next few years which were all managed with Diclofenac. Then 2.5 years ago you saw a visiting traditional healer at the Calgary Aboriginal health clinic who prescribed a brown medicinal tea that you mixed with warm water and drank every morning. You saw this healer every few months and got more tea and had no flares until the current one. You ran out of the tea a few months after moving to Toronto.

It took you a while to find a new family doctor when you moved to Toronto – but you heard that Dr. X (was accepting new patients there last year. You have seen Dr. X twice – once to meet them, and once with a bad bronchitis. You mentioned your ankylosing spondylitis but had no symptoms at either visit. You have also seen a walk-in doctor (who prescribed OTC Advil and ordered the bloodwork and X-ray) 3 days ago.

Dr. Barnabe did blood tests and X-rays of your cervical and lumbosacral spine following your first appointment and at your annual follow up appointments.

**TIMELINE OF THE PROBLEM**

**About 11 years ago**

- You began to experience lower back pain and stiffness that got progressively worse over several months

**10 Years ago**

- You were referred to Dr. Barnabe, a rheumatologist, by Dr. Lindsay Crowshoe, a family physician in the Aboriginal Health Clinic in Calgary.
- Dr. Barnabe did blood tests and X-rays of your cervical and lumbosacral spine following your first appointment and at your annual f/u appointments.
- After the results confirmed ankylosing spondylitis, a form of arthritis.
- Dr. Barnabe prescribed a course of Diclofenac to be taken only when experiencing a flare and your symptoms resolved quickly. It was also noted over the years that your Mantoux test is always positive because you received a BCG vaccination as a child

**Over the past 5 or 6 years**

- You have had 2 or 3 flares over the last 5 or 6 years.

**3 years ago**

- Your last flare for ankylosing spondylitis 3 years ago in Calgary.
- It was managed with Diclofenac that was prescribed by your Rheumatologist, Dr. Cheryl Barnabe
- You had chest x-ray that was clear

**Over the past 2 ½ years ago**

- You went to a traditional healer at the Calgary Aboriginal Clinic 2 ½ years ago who prescribed a brown medicinal tea that you mixed with warm water and drank every morning. You saw this healer every few months and got more of the tea and have not had any flares until the current one.

**1 year ago**

- You moved to Toronto from Calgary 1 year ago to be closer to your aging mother (72, lives in seniors’ complex, frail – complications of DM/widowed 5 years ago).

**About 10 months ago**

- You managed to find a new family doctor in Toronto who you’ve seen twice, once just to meet him/her and once for bad bronchitis.
- At the first visit you told the doctor about your ankylosing spondylitis, but had no symptoms during either visit. The doctor also took your blood pressure and it was noted that it was a bit high and you were told that you may have borderline hypertension. You were told to check it 3 times at the pharmacy and to try to reduce your salt intake and exercise regularly.
- Your new family doctor gave you a form to get blood work at your first appointment to check your kidneys and electrolytes because of your high blood pressure reading but you never went to get these blood tests

**6 months ago**:

- You ran out of the medicinal tea you received from the traditional healer you had seen

**2 months ago**

- Your lower back began to hurt and you recognized the pain from previous flares of your ankylosing spondylitis.
- The pain started subtly with stiffness and pain upon waking that got better when you moved around. It gradually progressed up your back and the pain and stiffness have now been waking you up at night. You started taking aspirin at the onset of this pain but it continue to progress.
- You have been taking 4-6 aspirin 325 mg per day over the last 2 months

**3 days ago**

- You went to see a walk-in clinic doctor close to your office for the pain from your ankylosing spondylitis flare
- It feels like you have a hot poker in your lower spine.
- The doctor who saw you to try extra strength advil three times a day and sent you for bloodwork and an X-ray of your lumbar spine and SI joints.
- You started taking the Extra strength Advil three times a day at the same time as you take the aspirin which is still averaging 4-6 325 mg tabs per day.

**2 days ago**

- You went to a lab in the morning and had blood work done and an x-ray done.

**Today**

- You had to call in sick to your work today as a bank manager as it was clear that you would not be able to sit for any length of time.
- The pain in your lower back is getting worse rather than better.
- You called the clinic today and were able to get an [urgent care/on-call appt]. Your regular family doctor is not in.
- You have spoken to your rheumatologist’s Dr. Barnabe’s office and have a pending referral with one of her Rheumatology colleagues in Toronto.
- You are currently unable to sit at all.
- For the past 24 hours you have been experiencing regular burning reflux within 20 minutes of taking the advil/aspirin. You don’t have any epigastric pain otherwise and your stools are normal.
- You have arrived with the empty Diclofenac bottle and are hoping that you will get a new prescription (note you don’t show the Diclofenac bottle to the triage nurse – you just show it to the nurse and physician in the treatment areas).
- You are also wondering where in Toronto you might be able to access a traditional healer.

**Full Scenario:**

You are **TOM/THERESA WHITFORD**, age 48, and are visiting this physician/NP for the first time. You moved to Toronto from Calgary 1 year ago to be closer to your aging mother (72, lives in seniors complex, frail – complications of DM/widowed 5 years ago). Your regular family physician, Dr. X, who you have seen twice since you arrived was not available when you called their office today.

Your presenting complaint is acute lower back pain. You have ankylosing spondylitis (diagnosed 10 years ago in Calgary). This pain is consistent with previous flares of your ankylosing spondylitis in that it started the same location (your lower back) and feels similar in nature. This time, the pain started subtly about 2 months ago with stiffness and pain upon waking that got better when you moved around. It gradually progressed up your back and the pain and stiffness have now been waking you up at night. You have been taking one or two aspirin, 325 mg 2-3 times per day since the pain started 2 months ago but it hasn’t helped. Three days ago you went to a walk-in clinic where the doctor told you to take extra-strength Advil three times a day and follow up with your family doctor if the pain persists. He also gave you requisitions for bloodwork and X-rays of your lumbar spine and SI joints which you had done. You have now added extra-strength Advil three times a day to the aspirin which you have continued to take. For the past 24 hours you have now been experiencing regular burning reflux within 20 minutes of taking the Advil/aspirin. You don’t have any epigastric pain otherwise and your stools are normal. You also called your rheumatologist’s office and a referral was made to a specialist in Toronto. You are waiting to hear back about an appointment. You don’t remember the name of the specialist.

You went to an outside lab and had the blood work drawn and X-ray done yesterday morning. You went online before coming into clinic today and printed out the results of these blood tests, which were all completed. Results of the X-ray were not yet available.

Since your saw the walk-in doctor 3 days ago the pain has been getting worse rather than better. Advil and aspirin are not helping. For the past 2 days it feels like you have a hot poker in your lower spine. You had to call in sick to your work today as a bank manager as it was clear that you would not be able to sit for any length of time. You are currently unable to sit at all without severe pain. You called the clinic today and were able to get an [urgent care/on-call appt]. Your regular family doctor is not in.

Your last flare was 3 years ago in Calgary and was managed with Diclofenac that was prescribed by your Rheumatologist, Dr. Cheryl Barnabe. 2.5 years ago you were prescribed a tea by a traditional healer that you saw at the Aboriginal health Centre in Calgary and you took this regularly and it prevented flares if you took it right away when you noticed symptoms but you ran out of this medicine six months ago. You have arrived with the empty Diclofenac bottle and are hoping that you will get a new prescription for Diclofenac since this worked well in the past. You are also wondering where in Toronto you might be able to access a traditional healer.

You are of First Nations descent (Cree, Saddle Lake FN – grew up in Calgary – mother moved to Toronto to be closer to the family of her second husband – your step-dad).

**MEDICAL HISTORY**

You had an appendectomy at age 10.

At your first visit to Dr. X (family doc) it was noted that your blood pressure was a bit high and you were told that you may have borderline hypertension. You were told to check it 3 times at the pharmacy and to try to reduce your salt intake and exercise regularly.

The bloodwork from yesterday shows a normal blood levels.

Your Mantoux test is always positive because you received a BCG vaccination as a child.

Chest X-ray films have always been clear.

The last was three years ago.

There is no history of gastric ulcer.

**MEDICATIONS**

You have been taking aspirin 325 mg 1-2 tabs 2-3 times a day for 3 months.

You have been taking over the counter extra-strength Advil three times a day as suggested by the walk-in doctor for pain but it doesn’t seem to be working and you are starting to experience a consistent burning reflux about 20 minutes after you take it.

You have your old Diclofenac bottle but need a new prescription.

You took a medicinal tea for arthritis prescribed by a traditional healer but ran out about 6 months ago.

**LABORATORY RESULTS**

As above, your new family doctor X gave you a form to get blood work at your first appointment to check your kidneys and electrolytes because of your high blood pressure reading but you never went to get these blood tests. You did go get the blood work ordered by the walk-in doctor at an outside lab yesterday morning and printed out these results and have brought them in today along with some of your old x-rays. You had an X-ray of your lumbar and SI spine but when you went on line those results were not yet available.

**ALLERGIES**

You are allergic to cats and penicillin. You had penicillin as a child and got a bad rash.

**IMMUNIZATIONS**

All childhood immunizations were done, including BCG. You think your family doctor in Calgary (Dr Crowshoe) gave you a tetanus shot and a pneumonia shot (Pneumovac) at one of your regular visits but that would be about 5 years ago.

You have not been getting flu shots because you are worried about the side effects.

**LIFESTYLE ISSUES**

**Alcohol:** You are an occasional social drinker. About 2-3 X per month and never more than 3 drinks at a time.

**Tobacco:** You smoke 6-10 cigarettes per day. You would like to quit but haven’t been able to.

**Illicit drugs:** In the past you have used marijuana, very rarely.

You last used it before you moved to Toronto

**Diet:** You try to eat well, but sometimes can’t resist fast foods and/or are too tired after work to cook.

**Exercise and Recreation:** You have no formal exercise program, but you

do walk to and from work, which is about 1 km each way. Since this flare you have been taking the subway or Uber since you are too sore to walk.

You have dinner with your mother every Sunday.

You enjoy watching hockey and have managed to get to a few Maple Leafs games since moving to Toronto.

You enjoy fishing, but haven’t been able to do this as much since moving to Toronto.

**FAMILY HISTORY**

You lost touch with your birth father as a teenager and have not tried to reconnect. He and your mother weren’t able to get along and he left your family when you were 12 years old.

Your mother remarried when you were 18 years old and moved to Toronto when you were 25 with your step-father so they could be closer to his family.

You have a younger brother, Jimmy Whitford, aged 46, who lives in Calgary and works as a roofer. He is healthy as far as you know.

Your maternal grandparents died in their late 60’s. Your grandfather went first with COPD and pneumonia. Your grandmother died a few months later from complications of a bad stroke. They both lived on Saddle Lake First Nations and you visited them occasionally when you were a child. You remember your grandmother was stooped over and had arthritis – but you don’t know if it was the same kind that you have.

**PERSONAL HISTORY**

You are heterosexual and currently single. You have had two long term relationships – the last one ended shortly before you moved to Toronto. You don’t have any children.

**EDUCATION AND WORK HISTORY**

You worked odd jobs and did some travelling after completing high school and then enrolled in college to study accounting. You successfully graduated at age 25. You started working at the CIBC in Calgary after college, reviewing lending applications. After several years you became branch lending manager, a position that you held for over 10 years in Calgary. Your regional lending manager in Calgary was able to support you in acquiring a branch lending manager position at a CIBC in Toronto, when you decided to move there.

**FINANCES**

You earn $70,000 per year in your job as a branch lending manager at CIBC.

You currently rent a bachelor apartment in Toronto. You own a small house in Calgary which you are renting out until the housing market in Toronto cools down.

**SOCIAL SUPPORTS**

You have made many acquaintances since moving to Toronto but have only a few closer friends and they are still in Calgary. In addition to having dinner with your mom every Sunday night you have a friend at work who is usually available to go watch a game at the local sports bar on Friday or Saturday.

You have been part of the Aboriginal Employee Circle at the CIBC for the past 10 years and are currently Vice-Chair.

**RELIGION**

You are not religious. You were raised Catholic by your mother but stopped going to church as soon as you could. You are resentful of the role of the church in residential schools.

You are interested in traditional spirituality but didn’t have much exposure since your mother was Catholic. You have a plan to visit your uncle who is a traditional ceremonialist in Saddle Lake and learn more. You remember attending a ceremony once with this Uncle during a summer visit to Saddle Lake (your mom had dropped you off with your grandparents for a month). You were comfortable sitting with the traditional healer in Calgary to talk about your arthritis pain and had been prepped by the healing coordinator to present him with tobacco. You miss your visits with this healer.

**EXPECTATIONS**

You expect that you will be given a prescription for Diclofenac.

You do not want repeat blood tests

You are hoping the physician might know of an Aboriginal health Centre where you could access a traditional healer and the traditional medicine that helped your symptoms.

You expect to be treated with dignity and respect.

**Progression of the scenario:**

- You will enter the urgent care clinic and register with the clerk who will be expecting you: provide your name, DOB, and address – tell them you are one of Dr. X’s patients.
- You will be asked to take a seat in the external waiting room.
- You will be called into the internal waiting room
- You will be seen by the physician

**Scenario Adaptations for Virtual UISP Visits Post-COVID**

In response to COVID-19 shutdown of non-emergency in-person clinic operations between March 2020 and September 2021, we pivoted to virtual UISP visits. Minor adaptations to the standardized patient scenario, UISP visit process, and UISP data collection methods were made as follows:

1. UISP visits were conducted through a hospital secured Zoom video call, which required the standardized patients to have access to high-speed internet in a private room. One standardized patient was unable to perform visits due to these requirements, while another who had previously relocated outside of the GTA was then able to perform visits remotely.
2. The scenario no longer included the standardized patient going to a walk-in clinic 3 days prior to the UISP visit. Instead, they visited a local pharmacy and attempted to refill their empty prescription of diclofenac. This pharmacist, instead of the walk-in clinic doctor, recommended taking Extra Strength Advil (400mg) three times daily.
3. A member of the study team posing as the local pharmacy faxed a fake diclofenac prescription renewal request to the clinic where the UISP visit was taking place. The unblinded site lead physician embedded the prop prescription renewal into the standardized patient’s EMR and ordered a follow-up consultation with the participant provider to occur via a virtual visit.
4. Previous in-person props, including the diclofenac pill bottle and laboratory blood test results, were not provided to standardized patients for the virtual visits. If the participant provider wanted to give the standardized patient any laboratory requisitions or prescription renewals, they were asked to fax them to a study-affiliated fax number.
5. Quality of Health Care Provider Relationship and Communication UISP assessment tool scoring prompts were adjusted to remove in-person quality markers of the patient’s experience. For example, when evaluating whether they were treated with respect and dignity, standardized patients were no longer asked to reflect on whether the participant provider left the room when they changed in/out of their clothes.
