# Supplementary material for: Randomized controlled trial demonstrates novel tools to assess patient outcomes of Indigenous cultural safety training
Source: BMC Med. 2024 Jan 9;22:3. doi: 10.1186/s12916-023-03193-y (PMC10775432; doi:10.1186/s12916-023-03193-y)
Supplement: Supplementary file 5 — Additional file 5. Supplemental Adjusted Model Results for Primary Outcomes and Supplemental Tables and Results for Secondary Explicit and Implicit Anti-Bias Outcomes. [file 12916_2023_3193_MOESM5_ESM.docx]

**Additional File 5**

Supplemental Adjusted Model Results for Primary Outcomes

For reference, full, adjusted multivariable regression model results for our primary outcomes (Table 3), including coefficients and standard errors are included below. Standard errors are in parentheses.

|  | Big Canoe | Little Canoe | Age  (in years) | Gender:  Women vs Men (reference) | Previous Indigenous Experience^b^ |
| --- | --- | --- | --- | --- | --- |
| Overall Patient Experience | 0.457  (0.167) | 0.398  (0.190) | -0.00444  (0.00596) | -0.0176  (0.158) | 0.0395  (0.0165) |
| Highly Recommend^a^ | 1.928  (0.904) | 2.052  (1.025) | 0.0434  (0.0309) | 0.545  (0.798) | 0.0831  (0.0789) |
| NSAID Renewal | -0.820  (0.759) | 0.606  (0.857) | -0.0569  (0.0268) | 0.0647  (0.732) | 0.0710  (0.0751) |
| Pain Management | 1.256  (0.738) | 1.540  (0.834) | -0.00526  (0.0261) | -0.591  (0.712) | 0.135  (0.0731) |

^a^ Multivariable logistic regression model; coefficients and standard errors are presented on the log-odds scale.

^b^ Likert scale (7 point)

Supplemental Tables and Results for Secondary Explicit and Implicit Anti-Bias Outcomes

Includes the Indigenous Implicit Association Test (IAT), Modern Prejudice Attitudes Towards Aboriginals Scale (M-PATAS), adapted from Morrison (1), Internal Motivation to Respond Without Prejudice Scale (IMS), and External Motivation to Respond Without Prejudice Scale (EMS), adapted from Devine & Plant (2)

These four secondary outcome measures were administered at baseline and following the unannounced Indigenous standardized patient (UISP) visits. Given the availability of baseline data, ANCOVA models were used to control for baseline scores and no further adjustments were made.

*Indigenous Implicit Association Test* results are scored between -2.0 and 2.0, where positive values denote a preference for white features, while negative values denote a preference for Indigenous features.

The *Modern Prejudice Attitudes Towards Aboriginals Scale (M-PATAS)* is comprised of 14 questions and scored on a 6-point Likert scale, where lower scores denote less prejudice. Both the Internal and External Motivation to Respond Without Prejudice Scale (*IMS*, *EMS*) scales are comprised of 5 questions scored on a 9-point Likert scale, with higher scores denoting more internal or external motivation, respectively. For the *M-PATAS, IMS* and *EMS* scales, average item results and statistics are presented to improve interpretation. Full survey questions are included at the end of this file.

Results:

The following supplementary tables present survey results as group means (SD):

|  | San’yas Intensive Training | Brief Anti-Bias Training | Control | ANCOVA  P-Value |
| --- | --- | --- | --- | --- |
| Implicit Association Test (IAT) |  |  |  |  |
| Baseline | 0.31 (0.41) | 0.25 (0.30) | 0.13 (0.50) | 0.399 |
| Follow-up | 0.26 (0.38) | 0.33 (0.34) | 0.12 (0.52) |  |
| N | 17 | 17 | 19 |  |
|  |  |  |  |  |
| Modern Prejudice Attitudes (M-PATAS)^a^ |  |  |  |  |
| Baseline | 1.74 (0.53) | 1.78 (0.46) | 1.53 (0.51) | 0.321 |
| Follow-up | 1.51 (0.43) | 1.68 (0.58) | 1.51 (0.46) |  |
| N | 19 | 17 | 17 |  |

^a^ M-PATAS scores are averaged over a 6-point Likert scale.

|  | San’yas Intensive Training | Brief Anti-Bias Training | Control | ANCOVA  P-Value |
| --- | --- | --- | --- | --- |
| Internal Motivation  (IMS)^b^ |  |  |  |  |
| Baseline | 8.3 (0.9) | 8.3 (0.8) | 8.6 (0.6) | 0.155 |
| Follow-up | 8.6 (0.6) | 8.3 (0.9) | 8.5 (0.7) |  |
| N | 19 | 17 | 17 |  |
|  | | | | |
| External Motivation  (EMS)^b^ |  |  |  |  |
| Baseline | 4.1 (1.5) | 3.9 (1.6) | 3.2 (1.4) | 0.250 |
| Follow-up | 3.9 (1.3) | 3.5 (1.8) | 3.9 (1.8) |  |
| N | 19 | 17 | 17 |  |

^b^ Internal and External motivation scales are averaged over a 9-point Likert scale

**IAT Example Tests**

**
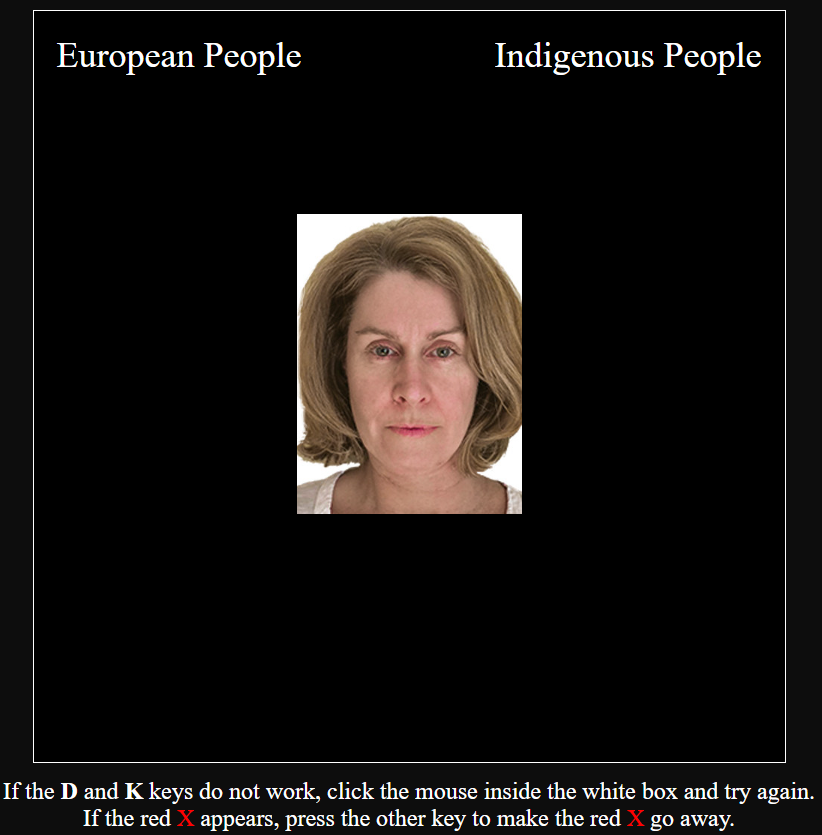
**

**
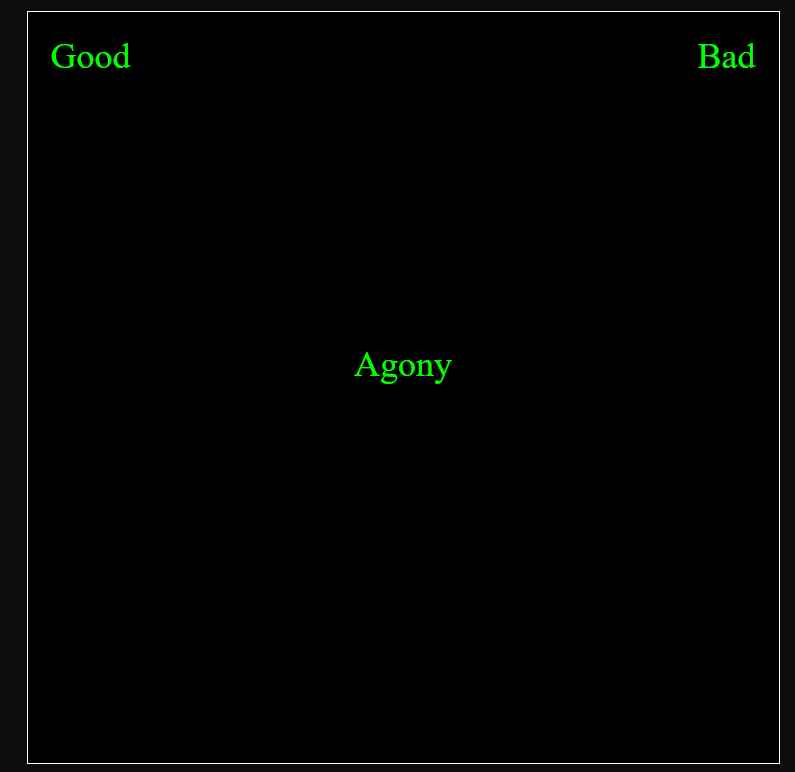
**

**
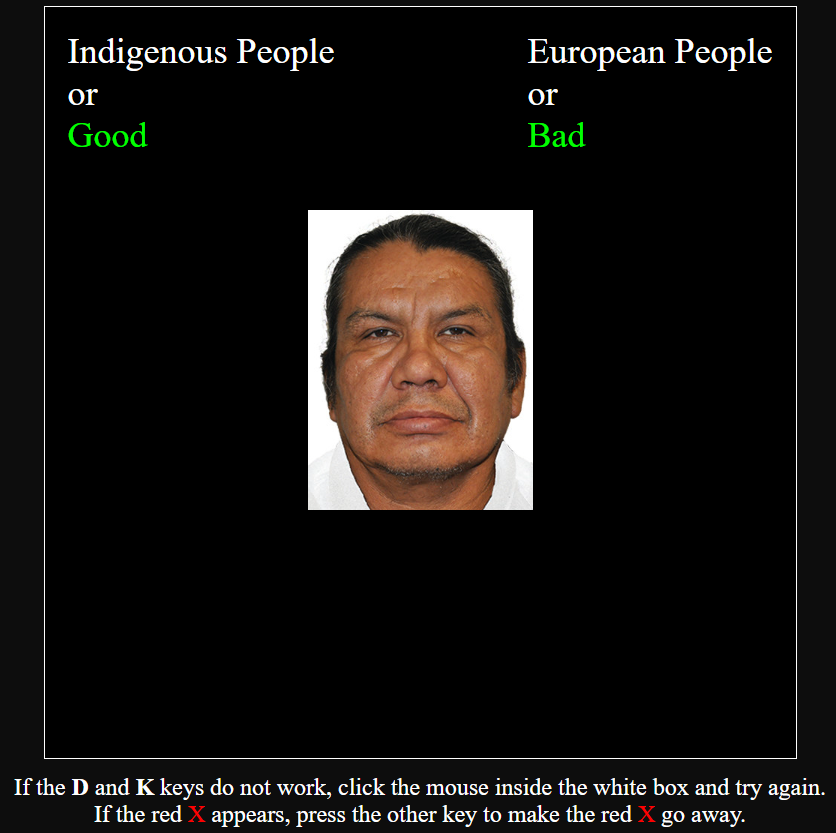

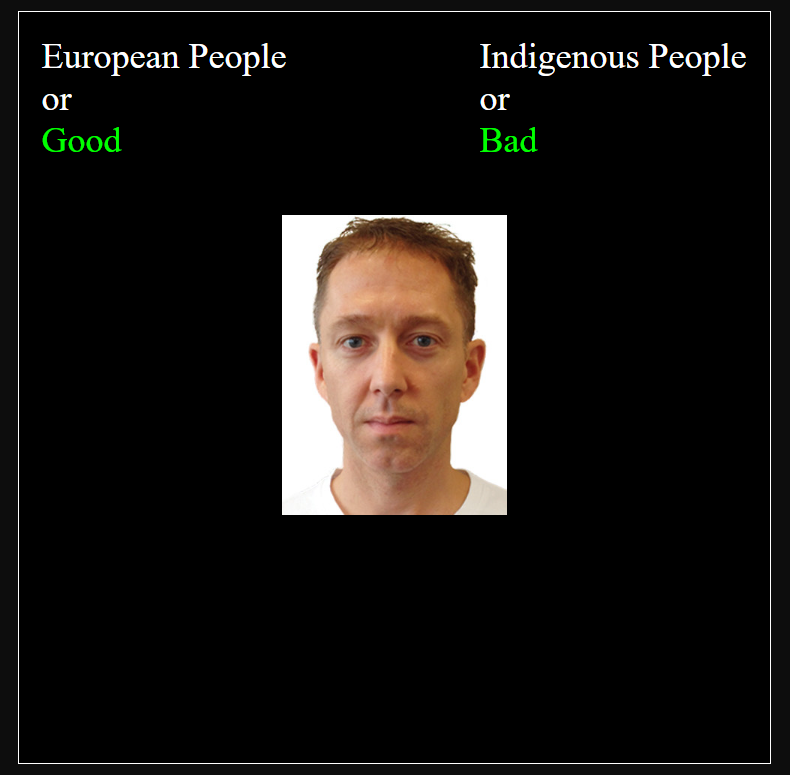
**

**Survey Questions**

*Modern Prejudice Attitudes Towards Aboriginals Scale (M-PATAS)*

| 1 | Canada needs to stop apologizing for events that happened to Indigenous people many years ago |
| --- | --- |
| 2 | Indigenous people still need to protest for equal rights* |
| 3 | Indigenous people should stop complaining about the way they are treated and simply get on with their lives |
| 4 | Indigenous people should get over past generations' experiences at residential schools |
| 5 | Indigenous people seem to use their cultural traditions to secure special rights denied to non-Indigenous people |
| 6 | Many of the requests made by Indigenous people to the Canadian government are excessive |
| 7 | Special places in academic programs should NOT be set aside for Indigenous students |
| 8 | Indigenous people should be satisfied with what the government has given them |
| 9 | It is now unnecessary to honour treaties with Indigenous peoples |
| 10 | Indigenous people should NOT have reserved placements in Universities unless they are qualified |
| 11 | Indigenous people should pay taxes just like everyone else |
| 12 | The government should support programs designed to place Indigenous people in positions of power* |
| 13 | Non-Indigenous people need to become sensitive to the needs of Indigenous people* |
| 14 | Government agencies should make every effort to meet the needs of Indigenous people* |

All variables coded on Likert Scale: (1:Strongly Disagree to 6:Strongly Agree)

*Indicates reverse coding

*Internal Motivation to Respond Without Prejudice Scale (IMS)*

| 1 | I attempt to act in non-prejudiced ways toward Indigenous people because it is personally important to me. |
| --- | --- |
| 2 | According to my personal values, using stereotypes about Indigenous people is OK. * |
| 3 | I am personally motivated by my beliefs to be non-prejudiced toward Indigenous people. |
| 4 | Because of my personal values, I believe that using stereotypes about Indigenous people is wrong. |
| 5 | Being non-prejudiced toward Indigenous people is important to my self-concept. |

All variables coded on Likert Scale: (1:Strongly Disagree to 9:Strongly Agree)

*Indicates reverse coding

*External Motivation to Respond Without Prejudice Scale (EMS)*

| 1 | Because of today's politically correct (PC) standards I try to appear non-prejudiced toward Indigenous people. |
| --- | --- |
| 2 | I try to hide any negative thoughts about Indigenous people in order to avoid negative reactions from others. |
| 3 | If I acted prejudiced toward Indigenous people, I would be concerned that others would be angry with me. |
| 4 | I attempt to appear non-prejudiced toward Indigenous people in order to avoid disapproval from others. |
| 5 | I try to act non-prejudiced toward Indigenous people because of pressure from others. |

All variables coded on Likert Scale: (1:Strongly Disagree to 9:Strongly Agree)

*Indicates reverse coding
