# Supplementary material for: Randomized controlled trial demonstrates novel tools to assess patient outcomes of Indigenous cultural safety training
Source: BMC Med. 2024 Jan 9;22:3. doi: 10.1186/s12916-023-03193-y (PMC10775432; doi:10.1186/s12916-023-03193-y)
Supplement: Supplementary file 7 — Additional file 7. Amount of Previous Indigenous Experience scale. [file 12916_2023_3193_MOESM7_ESM.docx]

**Additional File 7 - “Amount of Previous Indigenous Experience” scale:**

- 1. In the past, I have interacted with Indigenous people in many areas of my life (e.g. school, friends, work, clubs)
  2. The neighbourhood(s) I grew up in had mostly non-Indigenous people *
  3. The high school I attended had mostly non-Indigenous students *
  4. In the past, I have rarely interacted with Indigenous people. *

All variables coded on Likert Scale: (1:Strongly Disagree to 7:Strongly Agree)

*Indicates reverse coding

[Reference scale: Amount of Previous Experience with Black People, Plant & Devine, found in The Antecedents and Implications of Interracial Anxiety. Personality and Social Psychology Bullen. 2003;29(6):790-801]

Reference List:

1. Plant EA, Devine PG. The Antecedents and Implications of Interracial Anxiety. Personality & social psychology bulletin. 2003;29(6):790–801.
